# Iminosugar inhibitors of carbohydrate-active enzymes that underpin cereal grain germination and endosperm metabolism

**DOI:** 10.1042/BST20150222

**Published:** 2016-02-09

**Authors:** Vasilios M. E. Andriotis, Martin Rejzek, Michael D. Rugen, Birte Svensson, Alison M. Smith, Robert A. Field

**Affiliations:** * Departments of Biological Chemistry and Metabolic Biology, John Innes Centre, Norwich Research Park, Norwich NR4 7UH, U.K.; † Enzyme and Protein Chemistry, Department of Systems Biology, Technical University of Denmark, Elektrovej, Bldg. 375, DK-2800 Kgs Lyngby, Denmark

**Keywords:** arabinoxylan, cell wall, cereal grain, chemical genetics, iminosugar, starch

## Abstract

Starch is a major energy store in plants. It provides most of the calories in the human diet and, as a bulk commodity, it is used across broad industry sectors. Starch synthesis and degradation are not fully understood, owing to challenging biochemistry at the liquid/solid interface and relatively limited knowledge about the nature and control of starch degradation in plants. Increased societal and commercial demand for enhanced yield and quality in starch crops requires a better understanding of starch metabolism as a whole. Here we review recent advances in understanding the roles of carbohydrate-active enzymes in starch degradation in cereal grains through complementary chemical and molecular genetics. These approaches have allowed us to start dissecting aspects of starch degradation and the interplay with cell-wall polysaccharide hydrolysis during germination. With a view to improving and diversifying the properties and uses of cereal grains, it is possible that starch degradation may be amenable to manipulation through genetic or chemical intervention at the level of cell wall metabolism, rather than simply in the starch degradation pathway *per se*.

## Introduction

Starch, a glucose polymer, is of enormous biological and commercial importance. Starch, in roots, tubers and seeds, provides a carbon store for use by the plant during times when photosynthesis is not possible, such as regrowth following winter dormancy and during seed germination [1–5]. Starch also provides more than half of the calories in the human diet in the form of cereal grains (e.g. rice, maize, wheat, barley), roots and tubers (e.g. cassava and potato) and pulses (e.g. peas, beans). Additionally it is a valuable, versatile bulk commodity across the food, paper, textile and pharmaceutical sectors, as well as for bioethanol production [1,2]. Much research has been focused on the synthesis of starch in plants and the process is relatively well understood [2,6].

Starch degradation during grain germination is also of great societal and commercial importance, but has attracted less recent research. The success with which new generations of plants are established following germination and the vigour of the seedlings are largely determined by the interplay between an intricate developmental programme, genetically encoded and the supply to the growing embryo of energy from degradation of starch and other reserves in the seed [5,7]. The capacity for rapid and uniform emergence and for subsequent vigorous growth has profound implications for crop establishment, yield and hence commercial value. Starch degradation also underpins the brewing and distilling industries. Production of beverages from barley grains depends on establishing a balance between controlled increases in starch-active enzymes during the early stages of germination and seedling growth (malting), whereas at the same time minimizing loss of starch, the bulk of the fermentable material in the grain, due to degradation and seedling growth. Understanding starch metabolism and its regulation during cereal grain germination thus is important for improving starch crops both in terms of vigour following field germination and for biotechnological uses.

Molecular genetics in model species has provided significant insight into the enzymology and the regulation of starch degradation; however, studies of model plants alone are unlikely to provide sufficient information for crop improvement [1,2]. Here we discuss recent advances in understanding starch degradation during cereal grain germination. These have been facilitated through chemical genetics approaches, exploring the function of carbohydrate active enzymes in endosperm metabolism and in early seedling growth. Chemical genetics, an indispensable component of multidisciplinary systems approaches, offers substantial opportunity for discovery in seed biology and metabolism, relevant to crop improvement and global food security.

## Starch turnover

Starch consists of two polymers of α-D-glucose, amylopectin (a large polymer of α-1,4-linked chains, joined by α-1,6-branch points that occur in clusters at regular intervals along the axis of the molecule) and amylose (an essentially linear α-1,4-linked glucose chain) [1]. Adjacent linear chains form double helices that assemble into concentrically organized crystalline lamellae, interrupted by amorphous zones in which branch points occur at intervals of 9 nm. This semi-crystalline matrix is organized into higher-order structures, with periodicities of hundreds of nanometres. Starch is synthesized in plastids from ADP-glucose as the glucosyl donor via multiple isoforms of starch synthases, starch branching and debranching enzymes [1,6]. In leaves of many species, starch is synthesized during the light period in parallel with sucrose as a direct product of photosynthetic carbon dioxide assimilation. At night, starch is degraded to sustain respiration and maintenance processes in leaf cells and for conversion into sucrose for export to sink organs. In *Arabidopsis*, premature exhaustion of starch during the night or a block in starch degradation, leads to carbon starvation, resulting in large changes in gene expression and cellular metabolism and cessation of growth [8,9].

In heterotrophic tissues (e.g., cereal endosperm, cotyledons of beans and peas, cassava roots and potato tubers), starch is synthesized from sugars exported from source leaves [6,10]. Starch deposited in these organs is a long-term reserve for growth at specific times during plant development. For instance, in tubers and in perennial organs (e.g. roots) starch is degraded to sustain sprouting and growth following defoliation or dormancy [3,4]. In seeds and grains, it supports early seedling development until autotrophic growth is established [5].

**Figure 1:**
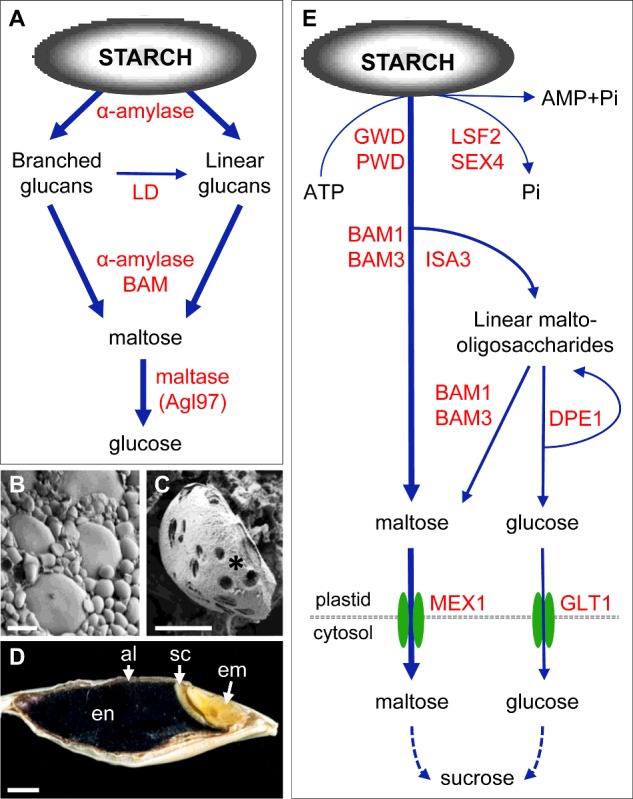
Starch degradation in cereal endosperm following grain germination and in *Arabidopsis* leaves at night (**A**) Pathway of starch degradation in cereal endosperm. (**B**) SEM of starch granules in mature barley grains. (**C**) SEM view of starch granule degradation (*) in barley grains 6 days post imbibition. (**D**) Tissue organization in barley grain. Shown is a longitudinal section through a barley grain, stained with iodine to visualize the starch deposits (black); al, aleurone layer; Em, embryo; en, endosperm; sc, scutellum. (**E**) Pathway of starch degradation in *Arabidopsis* leaves at night. Lesser fluxes involving α-amylase [1] are not shown for simplicity; GLT1, glucose transporter; MEX1, maltose transporter. Scale bars: (**B** and **C**) 5 μm, (**D**) 1 mm.

There is good reason to believe that the process of starch synthesis is conserved and it appears to be similar in leaves and storage organs (although the ratios of different classes of isoforms of starch synthase, starch branching and debranching enzymes vary between species and organs) [1,2,6]. By contrast, starch degradation proceeds by very different pathways in different types of organs [1]. The pathway of starch degradation in cereal grains is relatively simple (Figure 1A) and for long it was believed that the same pathway operates in other organs. Previous research suggests that this is not the case, e.g. it is now apparent that starch degradation in *Arabidopsis* leaves is different from the pathway established for cereal endosperm [1]. Re-examination of starch degradation in cereal endosperm in the light of these developments is revealing a complex picture and major questions remain to be addressed.

## Open questions about starch degradation in cereal endosperm

The current model of starch degradation in the endosperm of cereal grains involves four classes of glucoside hydrolases (Figure 1A). α-Amylase attacks the granule to produce a heterogeneous population of branched and linear glucans; branched glucans are converted into linear glucans by limit dextrinase (LD) and β-amylases (BAM) remove maltose units from the non-reducing end of linear glucans. The actions of α-amylase on linear glucans and of maltases (specialized α-glucosidases) on maltose, produce glucose for embryo growth. At the microscopic level, starch degradation is evident as pores on the granule surface (Figures 1B and 1C), progressively increasing internal erosion and granule disintegration.

Endosperm starch degradation is understood in great detail at the level of hormone action and gene expression, protein synthesis in the aleurone layer (a 2–3 cell layer surrounding the endosperm) and in the scutellum (a modified cotyledon; Figure 1D) and the structure–function relationships of several enzymes [11–14]. However, major questions remain about both the function and the specificity of most of the enzyme activities and the factors controlling the rate of supply to the embryo of sugars (e.g. glucose) from starch.

First, α-glucosidases may attack the starch granule directly or act synergistically with α-amylase to promote granule degradation [15]. It is unclear if barley Agl97, the major maltase in the grain, is the sole enzyme for maltose hydrolysis in the endosperm: the numbers, roles and properties of glucosidase isoforms of cereal grains remain unknown [16,17].

Second, there is ambiguity about the significance of both LD and BAM in the grain. Maize LD is necessary for normal rates of starch degradation early during kernel germination [18]. However, in barley newly synthesized LD is predicted to be targeted to the plastids of aleurone cells and not to the secretory pathway for entry into the endosperm [19]; its appearance in the endosperm is a late event during starch mobilization. The extent to which LD is actually active in the endosperm is also not clear since it is tightly bound by a proteinaceous inhibitor, LDI (LD inhibitor) [14,20]. The timing, localization and physiological importance of the interaction between LD and LDI are not fully understood. BAM produces much of the maltose generated in the endosperm (Figure 1A) [21], but it probably exerts little control over the production of sugars for embryo growth. For example, grains of BAM-deficient barley varieties and of rye mutants with low BAM activity germinate normally [22,23].

Thirdly, earlier research in *Arabidopsis* has identified new enzymes involved in leaf starch degradation [2]. Although it seems likely that the pathways of starch degradation in leaves and endosperm are different, the possibility that some of the newly-discovered enzymes are also involved in endosperm starch mobilization cannot be ruled out. In *Arabidopsis* leaves, starch degradation depends on the reversible phosphorylation of glucans at the granule surface through glucan and phosphoglucan water dikinases (GWD and PWD respectively) and the phosphoglucan phosphatases SEX4 (starch excess 4) and LSF2 (like SEX4 2; Figure 1E) [1]. This renders the granule surface more accessible to hydrolysis by BAMs and isoamylase 3 (ISA3). The major products of starch degradation in the chloroplast are maltose and glucose (from the action of the disproportionating enzyme (DPE1) on malto-oligosaccharides), which are exported to the cytosol for further metabolism [1,24].

## Complementing molecular genetics with chemical intervention

In *Arabidopsis*, molecular genetics have led to major advances in our understanding of leaf starch degradation at night [1]. However, these approaches have yielded little information on the enzymology of starch degradation in cereal endosperm during grain germination. In addition to the limited availability of genomic resources and the greater difficulty of cereal transformation, the application of molecular genetics is problematic because several enzyme activities for endosperm starch degradation are represented by multigene families and/or undergo extensive post-translational processing. Gene redundancy and partially overlapping enzyme specificities hinder prediction of phenotypic outcomes from loss-of-function mutagenesis. For example, in barley there are two families with a total of 10 genes encoding α-amylase and potentially four or more genes for α-glucosidase [16,17,25,26].

Systematic chemical intervention to evoke defined phenotypes offers a complementary approach to loss-of-function mutations. It involves either screening libraries of structurally unbiased compounds for phenotypic outcomes or targeted screens with small molecules interfering with specific enzyme functions (e.g. small molecule mimics of substrate or transition states in reactions catalysed by carbohydrate-active enzymes) [27]. As such, chemical genetics provide substantial opportunity for the dissection of multicomponent networks and complex biological processes, such as those associated with seedling growth and cereal endosperm metabolism.

Small-molecule inhibitors act at the protein level; they interfere with the function of products of multigene families with similar enzymatic mechanism, thus circumventing problems of redundancy and overlapping enzyme specificities [28]. Inhibitors may be tested at specific developmental stages for acute effects that can be both titrated and reversed. Thus, they provide temporal, conditional control over protein function, avoiding the lethality often observed when products of essential genes are targeted by genetic perturbation. Because of relative conservation of protein structure across species, chemical intervention approaches may be rapidly transferred between organisms (e.g. from *Arabidopsis* to oilseed and cereal crops; [29]).

Recent reviews provide extensive information on chemical genetics approaches in plants and other model systems [28–30]. Below we focus on new insights in grain physiology obtained through the application of small molecule inhibitors and the discovery of key regulatory processes underlying starch metabolism and seedling growth.

## Chemical genetics of endosperm metabolism in germinating barley grains

**Figure 2:**
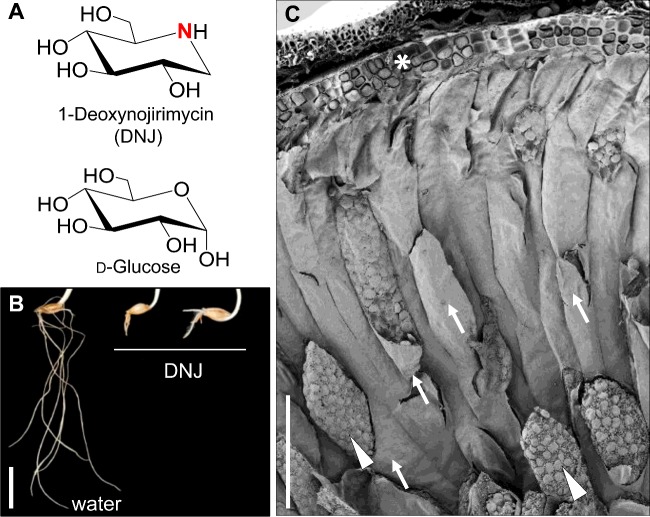
Iminosugar inhibitors of barley endosperm metabolism and root growth (**A**) Cemical structure of DNJ and D-glucose. The protonatable nitrogen within the ring of iminosugars is highlighted in red in the chemical structure of DNJ. (**B**) Root development in barley seedlings incubated from the point of imbibition with water (left) or DNJ (500 μM; right) for 10 days. (**C**) SEM view of a transverse section through the endosperm of a dry barley grain. Upon grain imbibition, many hydrolytic enzymes are synthesized in the aleurone cell layer (*) and scutellum and are then secreted into the endosperm. The endosperm is a non-living tissue. Endosperm cell walls (arrows) are a physical barrier to the diffusion through the tissue of hydrolytic enzymes, restricting their access to the starch deposits (arrowheads). Scale bars: (**B**) 1 cm, (**C**) 200 μm.

In order to understand the control of starch degradation in barley endosperm, we screened libraries of commercial drugs, bioactives and natural products for inhibition of recombinant barley Agl97 maltase activity *in vitro* [17]. These libraries included iminosugars, naturally occurring carbohydrate mimics in which a nitrogen atom replaces the endocyclic oxygen (Figure 2A). At physiological pH, the presence of protonatable nitrogen within the ring gives a positive charge to the molecule, thus mimicking the glucosyl cation intermediate of enzyme-catalysed glycoside hydrolysis [27,31]. This property makes iminosugars and their synthetic derivatives, potent inhibitors of a wide range of glucosidases, a fact broadly exploited in pharmacology [27]. For example, the iminosugar 1-deoxynojirimycin (DNJ), a D-glucose mimetic (Figure 2A), is a potent inhibitor of mammalian trehalase and of α- and β-glucosidases, glucosidases responsible for post-translational processing of asparagine-linked glycans and sucrose phosphate synthase [27,32–34].

Six-membered ring gluco-configured iminosugars (e.g. DNJ) were more potent inhibitors of Agl97 activity, than five-membered ring compounds, both *in vitro* and *in vivo* [17]. When applied to germinating barley grains they retarded root growth (Figure 2B) and the rate of starch loss in the endosperm. In contrast, grains of transgenic barley expressing an RNAi silencing cassette for *HvAGL97* had greatly reduced conversion of maltose into glucose but normal rates of starch loss in the endosperm and of root growth. Iminosugars and RNAi silencing of *HvAGL97* impair maltase function in the endosperm. These results imply that inhibition or loss of Agl97 by application of iminosugars or RNAi silencing impedes maltose metabolism as expected, but has no impact on starch loss as a whole. Therefore, iminosugars must target additional enzyme(s) necessary for normal rates of starch mobilization. These could be directly involved in starch degradation, for example, as-yet unidentified enzymes such as those discovered in *Arabidopsis* leaves (see discussion above; Figure 1). Alternatively, iminosugars may affect starch degradation indirectly; for example, they may target other processes in the endosperm that influence the rate at which starch is degraded.

One process that may be indirectly important for starch mobilization is cell wall degradation. The cereal endosperm (Figure 2C) is a non-living tissue, with no active transport mechanisms: following secretion from the aleurone layer and scutellum hydrolytic enzymes (e.g. α-amylase) must diffuse through the tissue to access the starch. Endosperm cell walls represent a physical barrier to the free diffusion of macromolecules through the tissue. In barley grain, they consist mainly of β-glucans (∼70% of cell wall material), arabinoxylan [AX; α-L-arabinofuranose (L-Ara*f*)-substituted β-(1,4)-xylan chains; ∼20%] and lesser amounts of cellulose and other polysaccharides [35,36]. It is widely believed that cell wall degradation, and hence permeabilization of the endosperm to macromolecules, occurs before starch mobilization [35]. Earlier work showed that during germination, cell-wall degrading enzymes diffuse in a wave-like pattern from the proximal (scutellum) end of the endosperm towards the distal end. The hydrolysis of cell wall polysaccharides spreads through the endosperm in a similar pattern [37]. The pattern of α-amylase diffusion and the spread of starch loss in the endosperm resemble the pattern of hydrolysis of cell wall polymers; thus starch degradation appears to occur immediately behind the front of cell wall degradation [37,38]. Despite these observations, the relationship between cell wall degradation and starch mobilization has not been tested experimentally.

To dissect the link between cell wall integrity and starch degradation, we undertook a chemical genetics approach focused on the hydrolysis of AX polymers. AX degradation to its constituent monosaccharides is mediated through the action of endo-1,4-β-D-xylan xylanohydrolases (EC 3.2.1.8; CAZy family GH10) which cleave the (1→4)-β-xylopyranosyl backbone of AX and AX arabinofuranohydrolases (AXAH; EC 3.2.1.55, CAZy family GH51) that release L-Ara*f* from the xylan backbone and also hydrolyse linkages between two arabinose residues. In addition, the exo-enzymes α-L-arabinofuranosidase and β-D-xylosidase also contribute to AX digestion by releasing terminal residues from oligosaccharides [39–43].

We found that particular iminosugars directly inhibit enzymes of AX degradation. When applied to germinating barley grains, they retard a wave-like spread of AX degradation through the endosperm and also retard starch loss over the same period (result to appear elsewhere). These findings strongly suggest that diffusion of macromolecules is restricted by endosperm cell walls; cell wall degradation is therefore required for normal rates of starch mobilization to occur. Our research provides new routes for genetic or chemical modulation of both the rate of cell wall mobilization and the rate of starch loss in the endosperm, with potential applications in the improvement of seedling vigour and the use of grains for ethanol production in the brewing and biofuel industries.

## Iminosugar inhibitors of root growth

The inhibition of barley root growth by iminosugars such as DNJ (Figure 2A) is also seen in wheat and *Arabidopsis* seedlings (V.M.E. Andriotis, A.M. Smith and R.A. Field, unpublished observations). This suggests that the inhibitors interfere with essential growth processes in the root common across plant species. Inhibition of root growth is not solely the consequence of slower mobilization of endosperm reserves and thus reduced availability of sugars for seedling growth [17]. DNJ also inhibits root growth when applied to isolated barley embryos; however, root growth is only partially restored by the provision of exogenous sugars. Therefore, iminosugars must have direct targets in the roots, in addition to those in the endosperm. Targets of iminosugars in the roots are currently unknown but they may include enzymes involved in root cell wall remodelling necessary for cell expansion. For example, in *Arabidopsis*, α-L-arabinofuranosidases (e.g. the bifunctional α-L-arabinofuranosidase/β-D-xylosidase ARAF1) are expressed in the root vasculature, in emerging lateral roots and in primary and secondary xylem in roots of adult plants [44,45]. Iminosugar targets may also be glucosidases I and II, which trim N-linked glycans during the maturation of glycosylated proteins in the endoplasmic reticulum. DNJ is known to inhibit these enzymes in plants [46]. Both glucosidase I and II have critical roles for normal plant development and growth. For example, in *Arabidopsis*, loss of glucosidase I in the *gcs1* mutant leads to embryo developmental arrest [47]. In addition, *rsw3* Arabidopsis mutant plants lacking glucosidase II α-subunit show radial root swelling, poor seedling development and a range of pleiotropic effects [48,49].

## Concluding remarks

We have highlighted recent advances in our understanding of starch degradation and cereal endosperm metabolism achieved through chemical genetics approaches. Our work provides new insights into endosperm metabolism and thus facilitates identification of new targets for informed breeding programmes aimed at cereal grain improvement for biotechnological applications and enhanced crop establishment and vigour in the field. The promiscuity of iminosugars (e.g. DNJ) [17,27,32] complicates interpretation of their effects during grain germination and seedling establishment; however, it provides a substantial opportunity to uncover mechanisms that control endosperm metabolism and seedling growth. We propose that multidisciplinary systems approaches in which chemical genetics and glycobiology tools (e.g. activity-based protein profiling using small molecule probes for the irreversible labelling of proteins in their active state; [50,51]) converge with molecular genomics, structural biology and genome editing [14,17,21,52], will be invaluable for the rational engineering of plant metabolism for enhanced seed quality and seedling vigour.

## Abbreviations

AX: arabinoxylan
BAM: β-amylase
DNJ: 1-deoxynojirimycin
L-Ara*f*: L-arabinofuranose
LD: limit dextrinase
LDI: limit dextrinase inhibitor
SEM: scanning electron microscopy
SEX4: starch excess 4
